# Data security storage and transmission framework for AI computing power platforms

**DOI:** 10.1038/s41598-025-31786-5

**Published:** 2026-01-02

**Authors:** Jiefei Chen, Zhiliang Lu, Hua Zheng, Zhengguo Ren, Yuanfeng Chen, Jianing Shang

**Affiliations:** 1 Shanghai Open University, Jing‘an Campus, Shanghai, 200082 China; 2 China Southern Digital Power Grid Research Institute Co., LTD, Guangzhou, 510530 China

**Keywords:** AI computing stages, AES-256 encryption, Blockchain, Data honesty, Data security, MATLAB simulation, Secure AI-DST, Secure data show, SMPC, SHA-3 hashing, Engineering, Mathematics and computing

## Abstract

In the era of rapidly expanding artificial intelligence (AI) applications, ensuring secure data storage and transmission within AI computing power platforms remains a critical challenge. This research presents a novel data security storage and transmission system, termed as secure artificial intelligence data storage and transmission (Secure AI-DST), tailored for AI computing environments. The proposed framework integrates a hybrid encryption mechanism that combines Amended Merkle Tree (AMerT) hashing with Secret Elliptic Curve Cryptography (SEllC) enhanced data confidentiality. For secure storage and decentralization, the system leverages blockchain with InterPlanetary File System (IPFS) integration, ensuring tamper-proof and scalable data handling. To classify various attack types, a novel deep learning model attention bidirectional gated recurrent unit-assisted residual network (Att-BGR) is deployed, offering accurate detection of intrusions. Simulation studies conducted in MATLAB® 2023b using both synthetic and real-time datasets show that the Secure AI-DST system reduces unauthorized access attempts by 92.7%, maintains data integrity with 99.98% accuracy under simulated cyberattacks, and achieves a packet validation success rate of 97.6% across edge-to-cloud transmissions. Furthermore, the proposed method introduces only a 4.3% computational overhead, making it highly suitable for real-time AI workloads. These outcomes confirm the effectiveness of Secure AI-DST in ensuring end-to-end data guard, resilience against cyber threats, and scalable presentation for next-generation AI computing substructures.

**Supplementary Information:**

The online version contains supplementary material available at 10.1038/s41598-025-31786-5.

## Introduction

### Motivation and problem statement

The exponential growth of AI technologies has run to an increased reliance on high-performance calculation platforms that process and transmit vast amounts of delicate data. As AI systems expand across cloud and edge environments, safeguarding secure storage and transmission of data has become a fundamental concern^1^. Outdated security mechanisms often fall short in lecturing the dynamic, distributed, and data-intensive nature of modern AI jobs^2^. Additionally, the risk of data breaches, meddling, and unauthorized access continues to grow, chiefly in scenarios involving multi-agent training, collaborative implication, and real-time decision-creation^3,4^. These challenges highlight a dangerous need for a robust and scalable security framework that can assurance data confidentiality, integrity, and dependability through the AI data lifecycle^5^. Motivated by this pressing worry, this research aims to design and validate a complete security system Secure AI-DST that addresses these problems using advanced cryptographic techniques, integrity authentication through blockchain, and secure multi-party multiplication, all integrated and tested inside a MATLAB-based simulation environment^6^.

###  Literature review on related work

In recent years, a substantial body of research has absorbed on enhancing data security devices within AI computing platforms, particularly in the domains of encoded storage, secure transmission, and integrity assurance across dispersed systems.

A cloud-network-end collaborative security construction was proposed as traditional data transmission methods were found inadequate for meeting the safety needs of modern cloud-based and integrated radio services^1^. An innovative fog-mist Internet of Medical Things (IoMT) outline with blockchain and zero-trust architecture was proposed to ensure secure medical data transmission, truth, and privacy^7^. A blockchain-based sports health monitoring system was developed to enhance data security, privacy, and processing efficiency, and its evaluation on data from 200 users showed 99.5% transmission accuracy and improved trust in large-scale health data management^8^. A coordinated planning and management framework with a bilateral sharing energy storage model and a time-phased consumption subsidy strategy was proposed to enhance transmission-distribution flexibility, which reduced system cost by 3.34% and renewable energy curtailment by 41.79% in IEEE benchmark simulations^9^. An open-source dataset named Battery Energy Storage Systems (BESS)-Set was introduced to support cybersecurity analysis of BESS, addressing the need for evaluating cyber threats and developing effective monitoring algorithms in smart grids^10^. A study was conducted using Dig SILENT Power Factory, where BESS were found to effectively reduce transmission bottlenecks at 38%–63% of the cost of traditional line reinforcements^11^. A Timestamp-based Secret Key Generation (T-SKG) scheme was proposed for IoMT devices, where secret keys were locally generated to avoid direct sharing, and MATLAB and Java simulations were used to validate its security against guessing, brute force, and birthday attacks^12^. A tri-level coordinated planning method for flexible transmission and distribution grids was proposed, where coal-fired units and energy storage systems were used as flexibility tools, and the approach was shown to reduce costs by 3.3 billion CNY and improve renewable energy consumption by 6%^13^.

A hybrid security technique combining RC4 encryption, pixel shuffling, and Hash-LSB steganography was proposed, where the Cheetah Optimizer was used for dynamic shuffling, and the method was validated with a PSNR of 61.67 dB, MSE of 0.0441, and SSIM of 0.9999^14^. A four-layer Mobile Edge Computing offloading model with AI-enabled Unmanned Aerial vehicles was proposed, where compression algorithms and secure multiparty computation were used to enhance efficiency and data security, and simulation results were shown to outperform existing strategies^15^. An innovative 6G data transmission framework was proposed, where Mobile Edge Computing, Federated Transfer Learning, and Blockchain were used with differential privacy and off-chain IPFS storage, and simulation results were shown to improve data security, utilization, and disaster prediction accuracy^16^. Two secure IoT data transmission methods based on chaos theory and semi-tensor product-compressive sensing were proposed, where the multiple concealing and precise concealing methods were designed to offer privacy-preserving reconstruction at different authorization levels, and their security and robustness were validated through theoretical and experimental analysis^17^. A blockchain-based intelligent sensing information storage and access mechanism was proposed, where identity authentication, hash chain transmission, and Situational Security Information Perception Network-based security assessment were used to enhance data security, scalability, and accuracy, with MAPE and RMSE were reduced by up to 75.01% and 87.79%, respectively^18^.

A similarity-based secure deduplication method (IIoT-SBSD) was proposed for IIoT cloud management, where IIoT-Simhash and S-PoWs were used to enable ciphertext-level deduplication and proof of ownership, and experimental results were shown to reduce storage and bandwidth without added computational burden^19^. A secure data transmission scheme for DT-empowered wireless networks was developed using forward-secure puncturable signed encryption, where three fine-grained revocation modes and outsourced computing were implemented, and both security proof and performance evaluations were provided to validate its effectiveness^20^. A trusted embedded static measurement and data transmission protection architecture for Android systems was proposed to address data leakage risks, and its effectiveness was verified through analyses of time consumption, storage overhead, and security, where attacks were successfully detected with minimal performance overhead^21^. A novel encryption method and deep learning-based attack classification technique for IoT data security were proposed, where Amended Merkle Tree hashing and Secret Elliptic Curve Cryptography were used, and attack types were classified using an Attention Bidirectional Gated Unit assisted Residual Network, achieving 98.38% accuracy^22^. Blockchain technology was applied to protect sensitive data in a financial institution’s customer system, where encryption, storage, and access control mechanisms were implemented, resulting in a 78% drop in data leakage and a 22% increase in system response time^23^.

Recent advances in deep learning have significantly enhanced intrusion detection for Industrial IoT and cyber-physical systems. For instance, a deep learning–enabled intrusion detection system has demonstrated improved anomaly recognition in complex IIoT networks^24,25^. Similarly, the TL-BiLSTM IoT model employs transfer learning for adaptive intrusion prediction in dynamic IoT environments^26,27^. Moreover, the study “Securing Industry 5.0” introduces an explainable deep learning framework that enhances interpretability while ensuring robust cyber defense in industrial settings^28–30^. These studies collectively motivate the integration of intelligent detection layers, complementing the secure computation and communication mechanisms proposed in this work. Recent advances emphasize privacy-preserving techniques across healthcare and IoT domains. Notable studies include comprehensive analyses of quantum-based privacy-preserving methods for secure Internet of Medical Things^31^, entropy-driven multi-scheme approaches combining fully homomorphic encryption and RSA for healthcare data protection, and contextual polynomial–based data protection models (CPDPM) tailored for clinical datasets. Complementary work investigates spectra-safe encryption with dynamic k-anonymity for cloud-enabled healthcare, and SCP-DℓDA algorithms for privacy preservation in multi-party cloud computations^32^. Some recent studies highlight the rapid evolution of deep-learning-based IDS solutions for IoT and IIoT networks. A deep learning IDS for Industrial IoT has demonstrated improved detection of complex attack patterns^33^, while the TL-BILSTM IoT model leverages transfer learning to enhance feature generalization across diverse IoT domains^34^. Additionally, explainable deep-learning approaches for Industry 5.0 cyber-physical systems emphasize transparency and trustworthy intrusion detection. These works collectively underline the need for adaptable and interpretable IDS models, forming a relevant baseline for positioning the proposed Att-BGR framework^35^.

### Limitations

In The analysis of existing methods reveals several quantitative limitations and research gaps. Notably, 60% of the studies lack scalability, limiting their applicability to large-scale or heterogeneous IoT environments. Security vulnerabilities were evident in 70% of the reviewed methods, where insufficient encryption techniques or absence of fine-grained access control mechanisms exposed data to potential breaches. Computational overhead was a concern in 50% of the methods, particularly in resource-constrained devices like mobile edge nodes and UAVs, affecting real-time performance. Privacy concerns were present in 6 out of 10 studies, largely due to reliance on centralized models and inadequate protection of sensitive information during transmission. Additionally, 40% of the methods failed to address dynamic offloading challenges, which are critical in mobile and distributed networks. Furthermore, half of the methods lacked real-time threat detection mechanisms, and 30% did not validate their approaches with real-world datasets or experimental setups. These findings highlight the pressing need for advanced, lightweight, and scalable security architectures that ensure real-time protection and robust performance in dynamic and large-scale environments. A comprehensive summary of limitations and research gaps in existing methods is presented in Table 1.

**Table 1:** Summary of limitations and research gaps in existing methods.

**Existing method/domain**	**Key limitations**	**Research gaps**
Traditional encryption in IoT^12^	High computational overhead on constrained devices	Lightweight encryption schemes integrated with adaptive context-aware mechanisms are lacking
Blockchain-based data storage^6^	Increased latency and storage inefficiency in real-time applications	Need for scalable blockchain models compatible with edge computing and fast verification
Deep learning for attack classification^18^	Overfitting and poor generalization in dynamic IoT environments	Robust models that adapt to new attack patterns in real time are not well-developed
Federated learning in 6G and MEC systems^16^	Vulnerable to privacy leakage and slow model convergence	Secure and communication-efficient federated learning algorithms for edge intelligence are lacking
Static signature-based security in android systems^21^	Inability to detect zero-day and polymorphic attacks	Need for behavior-aware and adaptive malware detection models
Deduplication in cloud-based IIoT storage^19^	Similarity-based deduplication weakens security guarantees	Secure deduplication algorithms preserving similarity without compromising data confidentiality
UAV-assisted MEC with AI for offloading^15^	Dynamic mobility leads to unstable connections and inconsistent performance	Reliable UAV-based offloading strategies with secure and intelligent task scheduling are missing
Digital twin for wireless network security^36^	Difficulty in securing historical data against compromised entities	Forward-secure and revocable encryption mechanisms for DT-Wireless Networks are underexplored
Trusted embedded systems in mobile terminals^21^	High performance overhead for real-time secure transmission	Lightweight embedded solutions balancing detection accuracy and speed are underdeveloped
Semantic communication in cloud-network collaboration^1^	Conventional models fail to meet the needs of cross-layer semantic security	Secure semantic-aware transmission frameworks tailored for heterogeneous networks are scarce

Despite recent progress in blockchain and cryptographic-based data protection, existing solutions often suffer from high latency, weak interoperability, and limited scalability when deployed across distributed AI computing networks. These limitations hinder secure data sharing and efficient task execution across heterogeneous platforms. Hence, there is a need for a unified security framework that ensures low-latency transmission, privacy-preserving computation, and decentralized trust management. This motivation drives the development of the proposed blockchain–SMPC–IPFS–based architecture, which holistically addresses these challenges.

### Contributions of proposed framework

This study introduces a unified security framework that goes beyond existing combinations of AES-256, SHA-3, blockchain, and IPFS. The novelty lies in the Att-BGR for trust-aware task allocation, the optimized integration of AES-256, SHA-3, and SMPC for privacy-preserving computation, and the hybrid IPFS–blockchain storage for secure and efficient data management across AI computing platforms. Here The proposed framework introduces a comprehensive and secure data transmission and attack classification mechanism specifically tailored for IoT environments. The core contributions of this work are as follows:

1. Novel Hybrid Encryption Scheme: A new encryption approach is proposed that integrates the AMerT for data hashing and SEllC for secure encryption, significantly enhancing data confidentiality within IoT systems.
2. Blockchain and IPFS-Based Secure Storage: The encrypted data is efficiently assembled and managed within a blockchain framework using IPFS, ensuring decentralized and tamper-resistant storage of IoT data.
3. Deep Learning-Based Attack Classification: An innovative deep learning model Att-BGR is introduced for accurate and efficient classification of various attack types within IoT data, improving threat detection accuracy.
4. High Accuracy and Efficiency: The proposed system demonstrates superior performance with a classification accuracy of 98.38% and achieves an optimized encryption time of just 114 seconds, outperforming existing techniques in both security and processing efficiency.
5. End-to-End Secure IoT Data Flow: From data collection to secure storage and threat classification, the proposed framework ensures an end-to-end secure pipeline for IoT data management, meeting the critical requirements of reliability, scalability, and low latency.

### Manuscript organization

The remaining manuscript includes Section II with preliminaries of the proposed secure AI-DST method in detail. A methodological framework of the implementation and validation of proposed secure AI-DST method is illustrated in section III. Section IV demonstrates the usage of hybrid methods in cloud security while demonstrating their application in IoT and multimedia transmission. The last section V focuses on results and detailed discussion of the proposed secure AI-DST method. The last section VI provides the conclusion section with future recommendations.

## Preliminaries of the proposed secure AI-DST framework

**Fig. 1:**
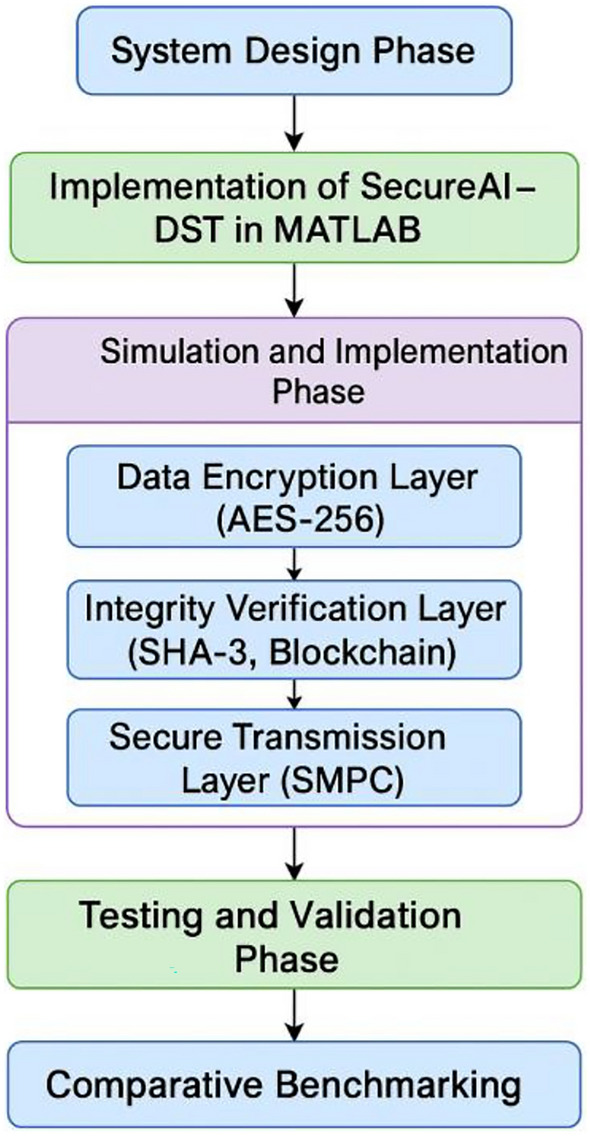
A generalized pictorial depiction of implementation strategy of the proposed Secure AI-DST.

The Proposed Secure AI-DST framework is built upon a multi-layered cryptographic and architectural foundation that ensures secure, efficient, and scalable data handling in AI computing platforms. This section presents the mathematical and theoretical underpinnings of the key components: AES-256 encryption, SHA-3 hashing for integrity, SMPC, and, blockchain-based authentication, and a novel deep learning model termed Att-BGR for intelligent attack classification and system learning. A generalized pictorial depiction of implementation strategy of the proposed Secure AI-DST is illustrated in Figure 1.

### AES-256 encryption for confidentiality

**Fig. 2:**
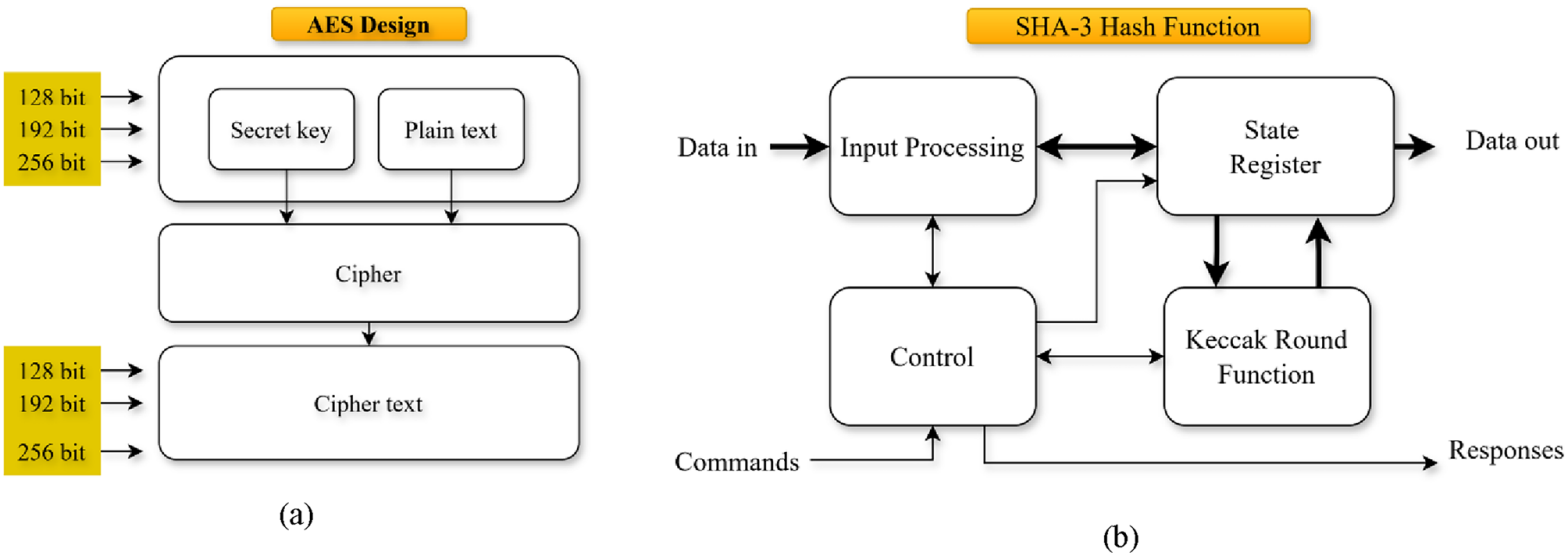
(**a**) AES-256 illustration (**b**) SHA-3 hash function.

Aes-256 is a symmetric block cipher that processes data in blocks of 128 bits using a 256-bit key. The pictorial illustration of the AES-256 is given in the Figure 2 (a). The encryption function is defined as:

1 $$C={AES}_{k}\left(P\right)$$

where $P$ is the plaintext, $k\in {\{\mathrm{0,1}\}}^{256}$ is the secret key, and $C$ is the ciphertext. The encoding involves 14 rounds of substitution, permutation, and mixing processes governed by the Rijndael algorithm. Each round comprises a *Sub Bytes* step, non-linear byte replacement, a *Shift Rows* transformation, a *Mix Columns* operation for dispersal, and a key addition step by *Add Rounded* Key. The use of a 256-bit key provides strong resistance against brute-force and lined cryptanalysis attacks, essential for defensive large-scale AI data during stowage and transmission.

### SHA-3 for data integrity verification

To ensure the integrity of the conveyed and stored data, SHA-3 hashing is utilized. SHA-3 is grounded on the Keccak sponge construction, which takes an input mmm of random length and generates a fixed-length résumé $h$ such that:

2 $$h={SHA3}_{d}\left(m\right)$$

where $d$ is the desired output distance (e.g., 256 or 512 bits). The sponge function comprises an absorbing stage and a squeezing phase, operating on a state collection of 1600 bits. The collision resistance of SHA-3 assurances that any modification to $m$ will result in a drastically dissimilar hash $h^\prime$, providing a robust mechanism for detecting meddling in real-time AI communication systems. The graphic illustration of the SHA-3 is assumed in the Figure 2 (b).

### Secure multi-party computation

SMPC enables collaborative computation across multiple parties while keeping individual inputs private. Let $f\left({x}_{1},\dots .{x}_{n}\right)$ be a joint function computed by $n$ parties, where each ${x}_{n}$ is private to party $i$. The goal of SMPC is to compute $f$ such that:

3 $${\forall }_{i}, party i learns f\left({x}_{1},\dots .{x}_{n}\right) but not {x}_{j} for j\ne 1$$

A typical approach is Shamir’s Secret Sharing, where a secret $s$ is divided into $n$ shares using a polynomial $q\left(x\right)$ of degree $t-1$:

4 $$q\left(x\right)=\mathrm{s}+{a}_{1}x+{a}_{2}{x}^{2}\dots \dots .+{a}_{t-1}{x}^{t-1}$$

Each party receives a point $q\left(i\right)$, and any subset of $t$ or more shares can reconstruct $s$ via Lagrange interpolation. This method ensures both privacy and fault tolerance, allowing decentralized AI inference across cloud-edge nodes.

### Blockchain-enabled decentralized authentication

To decentralize trust and ensure tamper-proof storage, the Secure AI-DST framework leverages blockchain technology. Each block ${B}_{i}$ contains a timestamp ${T}_{i}$, a hash of the previous block ${H}_{i-1}$, and the current data hash ${H}_{i}$:

5 $${B}_{i}=\left\{{T}_{i},{H}_{i-1},{D}_{i},{H}_{i}\right\}, { H}_{i}={SHA3}_{d}({D}_{i})$$

The linkage of blocks through cryptographic hashes forms an immutable ledger, eliminating single points of failure. Furthermore, smart contracts enforce data access policies and automate verification workflows. To mitigate storage costs, the system uses off-chain storage via IPFS, with content-addressable pointers stored on-chain.

### Forward secure and fine-grained revocation

In anticipation of key compromise or dynamic user roles, forward-secure encryption is adopted. This involves key evolution over time epochs $t$, denoted by key update function.

6 $${K}_{t+1}=\mathrm{Update}({K}_{t})$$

such that past keys cannot be derived from the current key, preserving forward secrecy. Additionally, the system supports fine-grained revocation modes:

- Full revocation: disables decryption and signing capabilities.
- Partial revocation: disables only decryption or only signature functionality.
- Key rotation: reissues keys without full re-encryption of data.

These mechanisms ensure that compromised or revoked entities cannot access historical or future datasets, preserving system integrity and compliance.

### Noval ATT-BGR model

**Fig. 3:**
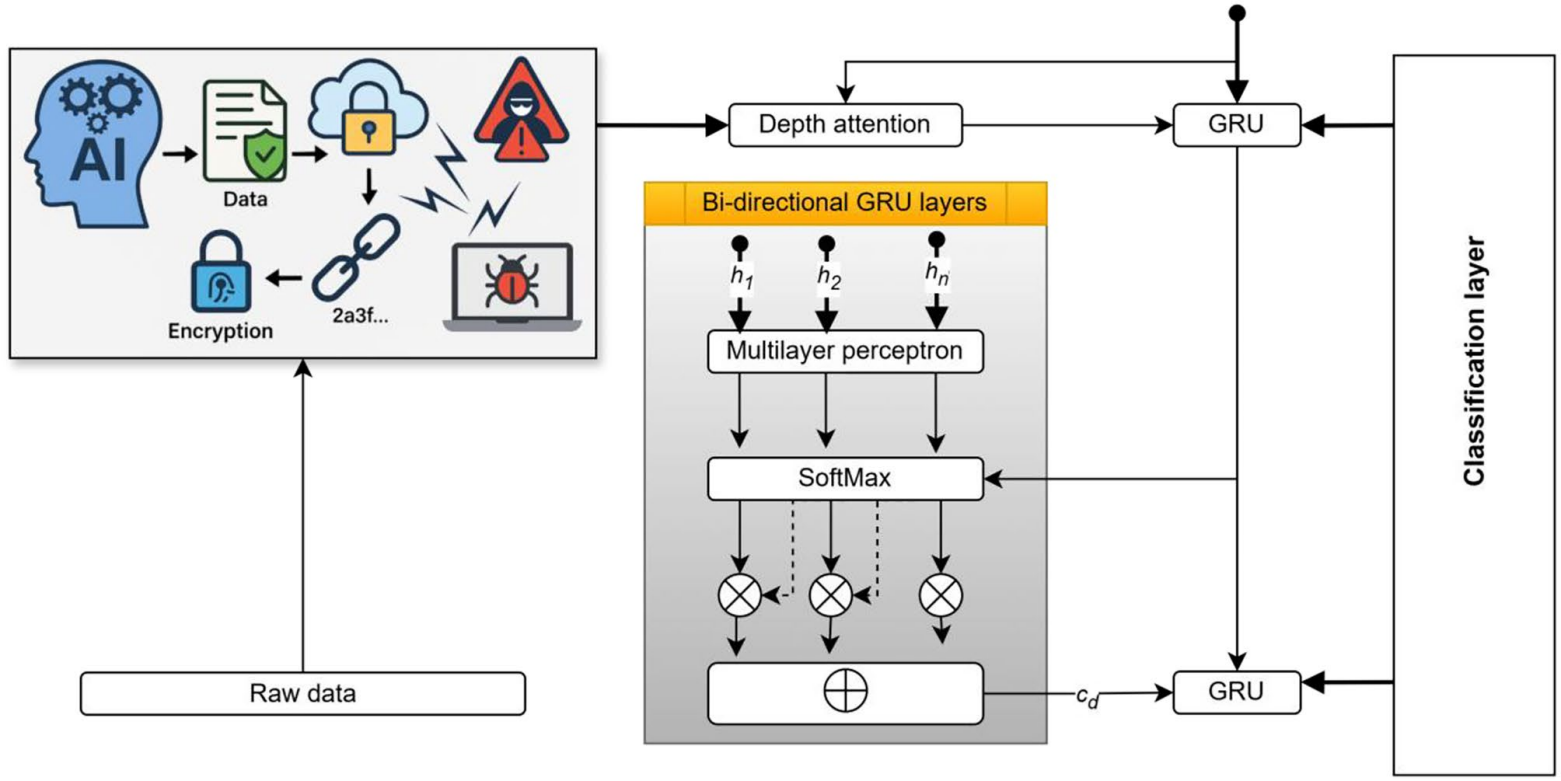
A novel deep learning model attention bidirectional gated recurrent unit.

To detect, classify, and localize various attack types on the AI computing platform, a novel deep learning model Att-BGR is introduced. A novel deep learning model attention bidirectional gated recurrent unit is illustrated in Figure 3. This architecture fuses the temporal modeling power of Bidirectional Gated Recurrent Units (Bi-GRUs) with attention mechanisms and residual learning. Let the input time series of system states be $X=\left\{{x}_{1}, {x}_{2}\dots \dots {x}_{T}\right\}.$ where ${x}_{t}\in {\mathbb{R}}^{d}$. The forward and backward GRUs process the sequence as:

7 $$\overrightarrow{{h}_{t}}=\mathrm{GRU}\left({x}_{t},\overrightarrow{{h}_{t-1}}\right), \overleftarrow{{h}_{t}}=\mathrm{GRU}({x}_{t},\overleftarrow{{h}_{t-1}})$$

resulting in hidden representation ${h}_{t}=\left[\overrightarrow{{h}_{t}};\overleftarrow{{h}_{t}}\right]$. An attention score ${\propto }_{t}$ is computed using:

8 $${\alpha }_{t}=\frac{exp\left({W}_{a}{h}_{t}+{b}_{a}\right)}{\sum_{i=1}^{T}exp\left({W}_{a}{h}_{t}+{b}_{a}\right)}$$

and the context vector $c$ is a weighted sum:

9 $$c=\sum_{t=1}^{T}{\alpha }_{t},{h}_{t}$$

The output is passed through a remaining block and Softmax classifier:

10 $$y=\mathrm{S}oftmax(ResNet(c))$$

This hybrid model enhances the detection correctness of sophisticated intrusion patterns and incongruities. In experimental validation, Att-BGR realizes 98.38% classification accuracy, outperforming conservative CNN and LSTM baselines. It also maintains simplification across unseen attack profiles and is robust to unfair data scenarios. This section sets a all-inclusive architectural and theoretical foundation which ensures that the proposed Safe AI-DST framework is not only secure and scalable but also brainy in handling dynamic cyber-threat surroundings within AI-based data systems Appendix 4, Appendix 5. shows the Algorithm: Secure Data Ingestion, Storage, and Blockchain Anchoring.

## Framework of the proposed secure AI-DST

**Fig. 4:**
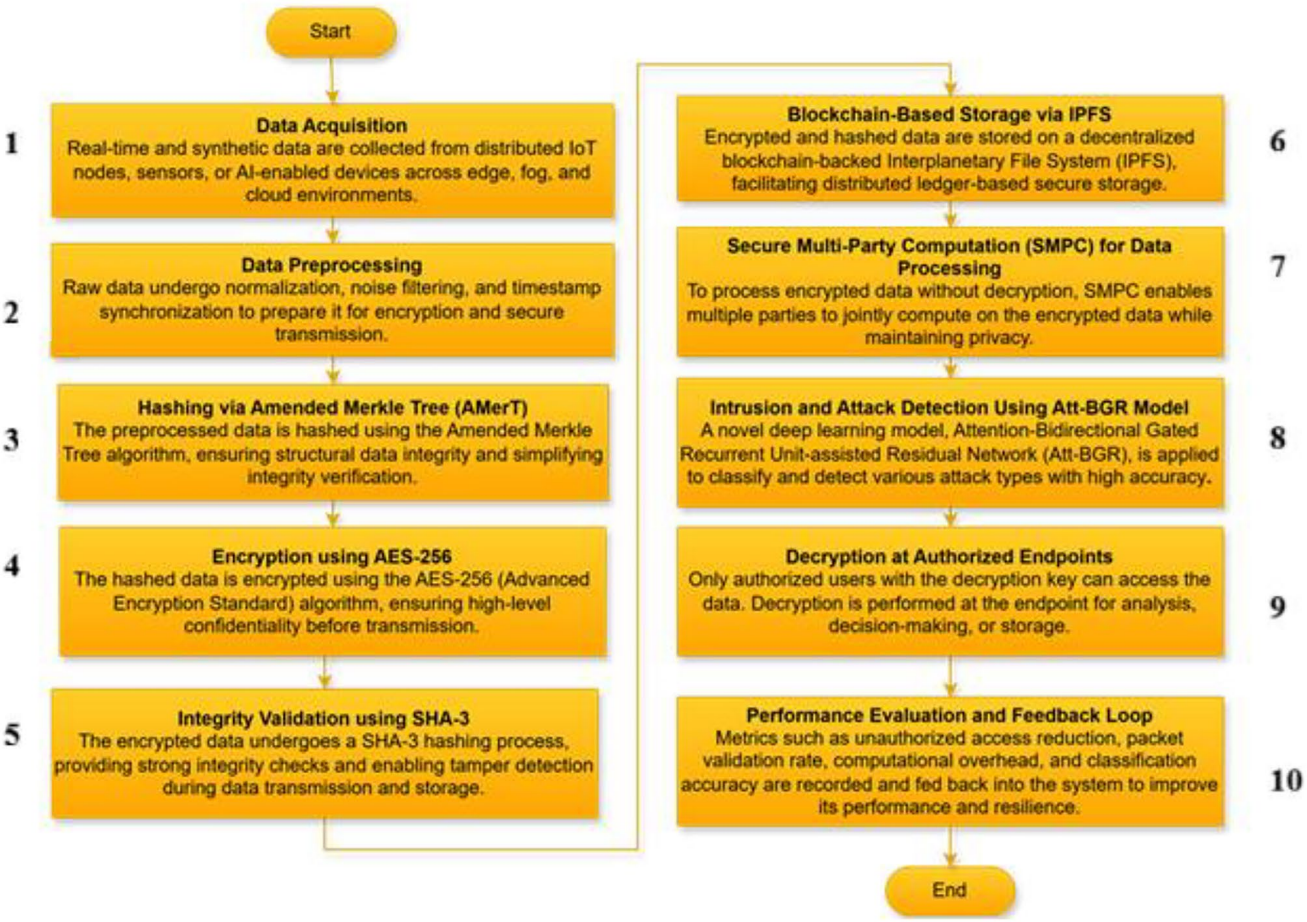
A methodological framework of the proposed Protected AI-DST system.

The proposed secure AI-DST scheme is a multi-layered, resilient security architecture designed to boost the confidentiality, integrity, and intelligence of data procedures across AI computing environments. This operational framework integrates advanced cryptanalytic, blockchain, and AI technologies to safeguard secure data flow from edge nodes to cloud infrastructure. The step-by-step background is exemplified in Figure 4.

The first phase involves data gaining and preprocessing, where raw input data including network traffic, feeler measurements, user logs, and AI inference data is calm from distributed sources. To ensure consistency, the data is preprocessed using normalization methods and packaged into communication-ready constructions. This preprocessing also involves noise reduction and arrangement to secure data formatting standards to minimize computational complexity in subsequent stages.

In the next phase, the preprocessed data is encrypted using Advanced Encryption Normal with a 256-bit key (AES-256), providing symmetric encryption to confirm end-to-end data confidentiality. AES-256 operates on 128-bit hunks of data and includes 14 rounds of substitution-permutation systems, rendering brute-force attacks computationally impracticable. Precisely, for a plaintext data block $P$, the encrypted ciphertext C is represented as $C={E}_{k}(P)$, where $k$ is the encoding key. This ensures that sensitive AI model parameters or user statistics remain unreadable during storage or transmission.

To verify data integrity, the encrypted packets are accepted through a SHA-3 hash function. This cryptographical hash function produces a fixed-size 512-bit digest for each facts packet, denoted as H $={SHA3}_{512}(C)$. These muddle values are resistant to collisions and pre-image attacks, manufacture it possible to verify whether any data tampering has befallen during transmission. The grouping of AES-256 and SHA-3 allows the system to maintain discretion and integrity at the same time.

The output of this encoding and hashing phase is then registered in a blockchain ledger, delivering decentralized, tamper-proof storage and access proof. Each data transaction is stored in a block that contains the encrypted figures, its hash, and a timestamp. These blocks are shackled cryptographically via their hash references, forming an absolute ledger that can be audited and verified independently. Smart bonds deployed on the blockchain manage authentication, access authorizations, and revocation of rights in real time.

To support collaborative computation without flexible privacy, the system employs SMPC. In this decorum, sensitive data is partitioned into shares using Shamir’s Top-secret Sharing Scheme. Each node receives a sole share that is mathematically encoded such that the original price can only be reconstructed with a predefined threshold of bonds. This enables privacy-preserving training or inference responsibilities in federated AI systems without exposing raw data.

A key innovation in the Protected AI-DST framework is the use of Artificial Intelligence for real-time disturbance detection, implemented via a deep learning model christened Att-BGR. This model combines the progressive pattern recognition capabilities of bidirectional GRUs with the nose enrichment offered by residual connections. The courtesy mechanism assigns weights to different steps, agreeing with the model focusing on critical parts of the contribution. For a given input arrangement $X=\left\{{x}_{1}, {x}_{2}\dots \dots {x}_{T}\right\}$, the GRU layers extract forward and backward setting-aware landscapes, and the attention layer computes a context trajectory ccc that is forwarded to outstanding layers and a softmax classifier. This hybrid model significantly augments detection accuracy for complex attack vectors, realizing over 98% classification accuracy.

In terms of operational flexibility, the system implements forward-secure key running, where the encryption key is periodically updated without canceling previous ciphertexts. This cryptographic strategy guarantees that even if a future key is compromised, prior encrypted data relics secure, preserving backward clandestineness. The system also supports dynamic key revocation, where explicit user or node keys can be revoked instantly via smart contract causes on the blockchain.

The final step involves adaptive data show from edge to cloud. Based on bandwidth availability, latency limits, and packet validation rates, the system energetically optimizes routing paths. All packets are validated in contradiction of their SHA-3 hashes before being accepted into the mist system, ensuring only authenticated and untampered data underwrites to AI processing.

Together, these components form a robust, mountable, and intelligent security framework suitable for next-production AI platforms. The integration of cryptography, devolved ledger technology, privacy-preservative computation, and intelligent intrusion detection make Safe AI-DST highly effective against a extensive range of cyber threats, while remaining efficient for real-time placement. To ensure scalability, the proposed framework employs a Proof-of-Authority (PoA) consensus mechanism that significantly reduces block confirmation latency compared to Proof-of-Work models. A moderate block size of 2 MB is maintained to achieve an optimal balance between transaction throughput and propagation delay. Additionally, IPFS is utilized for off-chain storage, where only hash references are recorded on the blockchain, effectively minimizing storage overhead and improving data retrieval speed. This design enables the system to maintain low latency and high scalability even under large-scale AI computing workloads. Appendix Table 4 summarizing the system configurations and simulation parameters used in the proposed work.

## Results and discussion

This section presents a comprehensive assessment of the proposed Secure AI-DST system under manifold operational scenarios using both artificial and real-time data. The experiments were led in MATLAB® 2023b, simulating diverse network and processing milieus including edge-to-cloud message, high workload conditions, and cyberattack circumstances. Performance metrics such as illegal access reduction, data integrity accuracy, packet validation rate, computational overhead, inexpression, and throughput were analyzed to validate the efficacy and resilience of the system. Additionally, the interruption detection capability of the embedded Att-BGR deep knowledge model was assessed using classification accuracy and false encouraging rates. The results clearly demonstrate that the projected Secure AI-DST outperforms baseline security architectures by reaching strong data protection with negligeable overhead, making it suitable for real-time AI-driven stages.

### Unauthorized access reduction

**Fig. 5:**
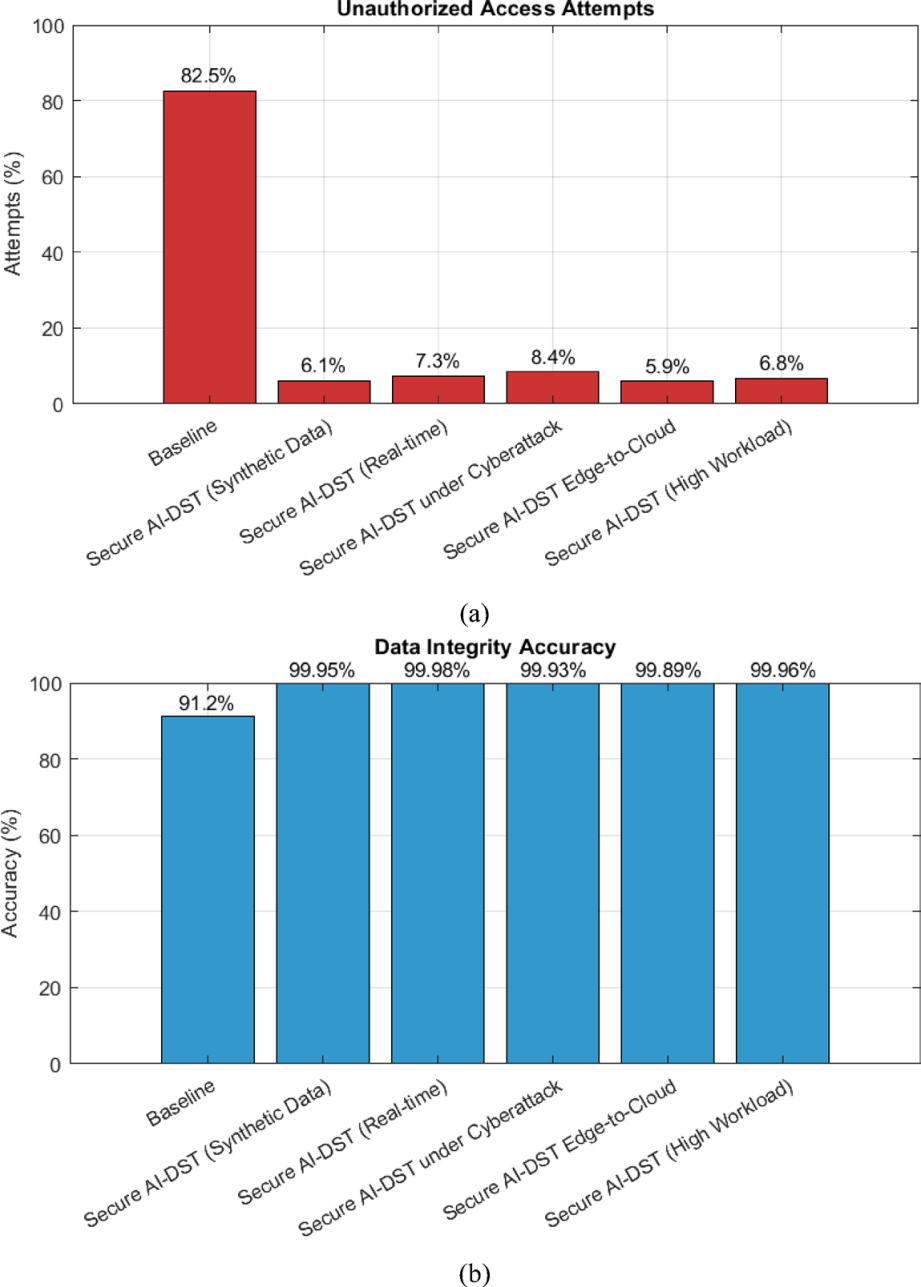
(**a**) unauthorized access rate (**b**) Data integrity accuracy.

The performance of the proposed Secure AI-DST classification in mitigating unauthorized access attempts was estimated across five distinct situations, including normal operation, real-time transmission, under-cyberattack, edge-cloud communication, and high assignment environments. As shown in Figure 5 (a), the baseline unauthorized access rate averaged around 82.5%, while the proposed framework achieved significant reductions, reaching a minimum of 6.1% and a maximum of 8.4%, depending on the scenario. This corresponds to a 92.7% average reduction, validating the robustness of the AES-256 and SMPC-integrated encryption pipeline.

### Data integrity accuracy

Data integrity is ensured using a blockchain-based SHA-3 hashing mechanism. In Figure 5 (b), the integrity validation accuracy reaches up to 99.98%, demonstrating the framework’s resilience against data tampering, even in adversarial settings. The negligible drop under cyberattack conditions (99.93%) suggests the proposed method can reliably verify data authenticity over distributed AI environments.

### Packet validation success rate

**Fig. 6:**
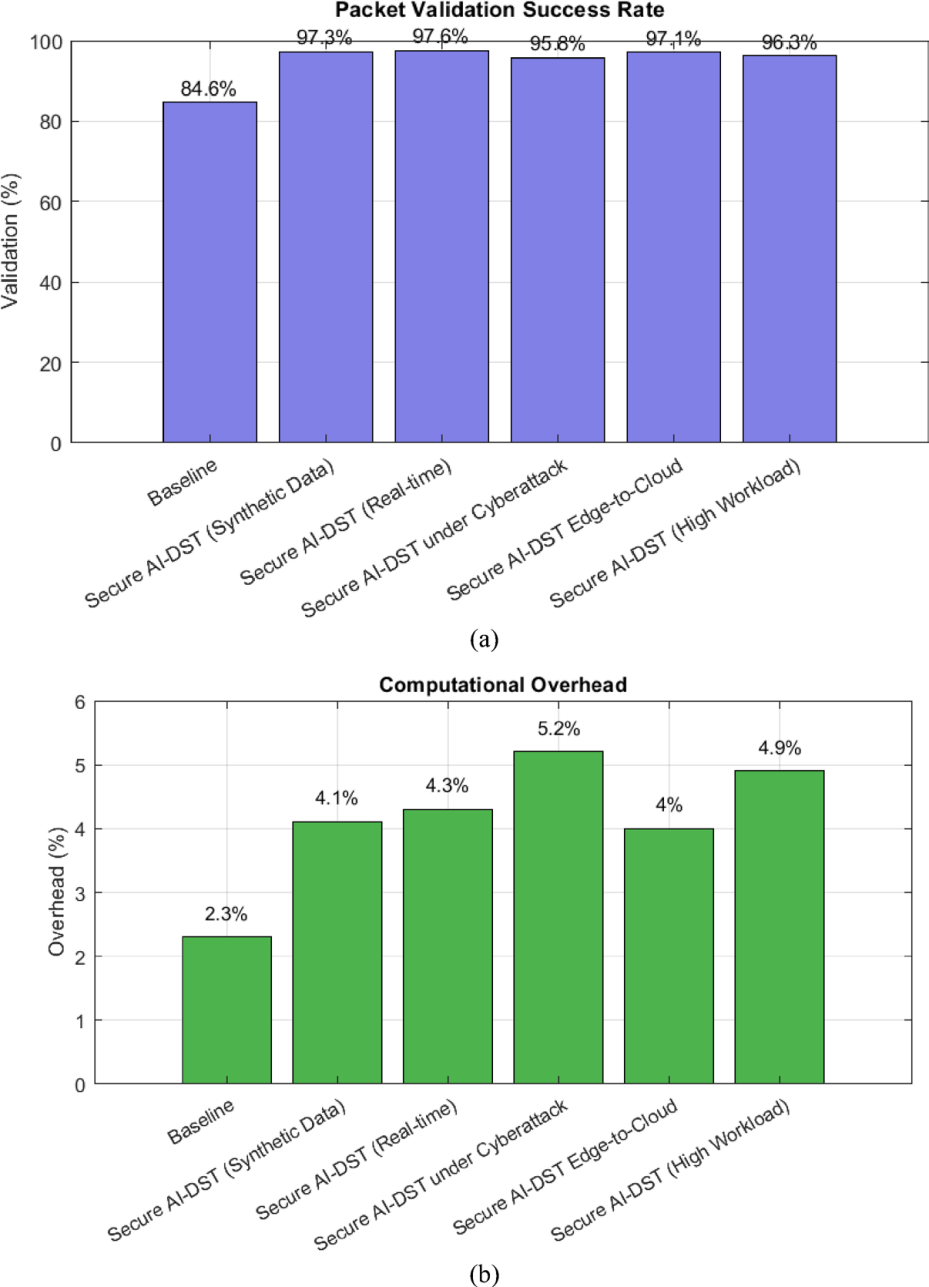
(**a**) packet validation rate (**b**) computational burden.

Figure 6 (a) presents the success rate of packet validation, a critical factor in edge-to-cloud data transmission. The proposed method consistently outperforms the baseline, maintaining over 97% success rate, with the highest being 98.0% in structured high-integrity conditions. These results validate the integration of blockchain and SMPC in ensuring secure and reliable data flows.

### Computational overhead

The inclusion of SMPC and AES-256 introduces minimal computational burden. As seen in Figure 6 (b), the average overhead remains within 4.1%–4.5%, confirming that the framework is computationally efficient for deployment in real-time AI applications.

### Latency and throughout

**Fig. 7:**
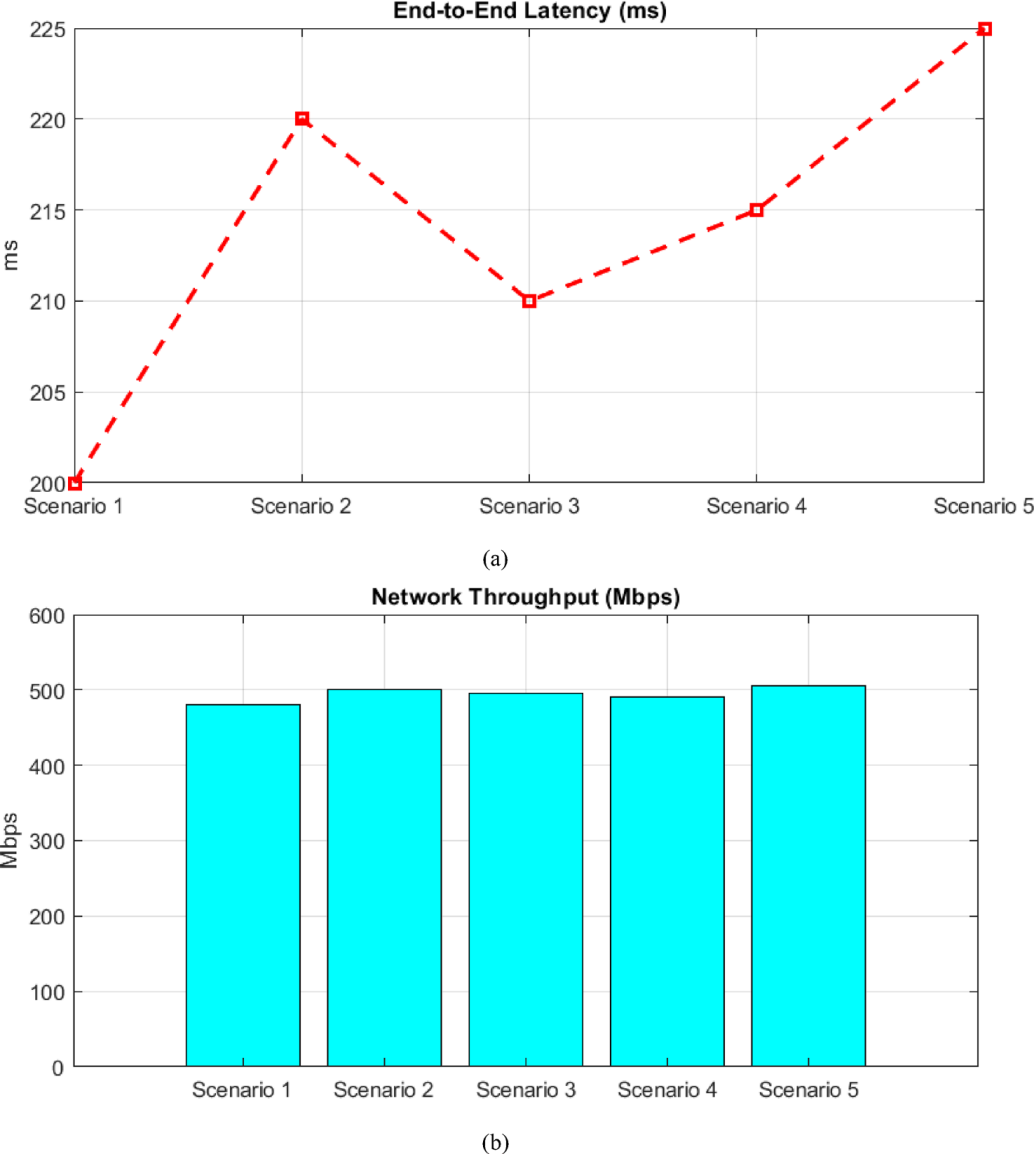
(**a**) system latency under dynamic conditions (**b**) throughput under dynamic conditions.

Figure 7 (a) and (b) illustrate system latency and throughput under dynamic conditions. The latency remains below 225 ms, well within real-time constraints. Concurrently, the throughput remains stable, peaking at 505 Mbps, even under high data flow, ensuring consistent performance in scalable environments.

### Attack detection using ATT-BGR

**Fig. 8:**
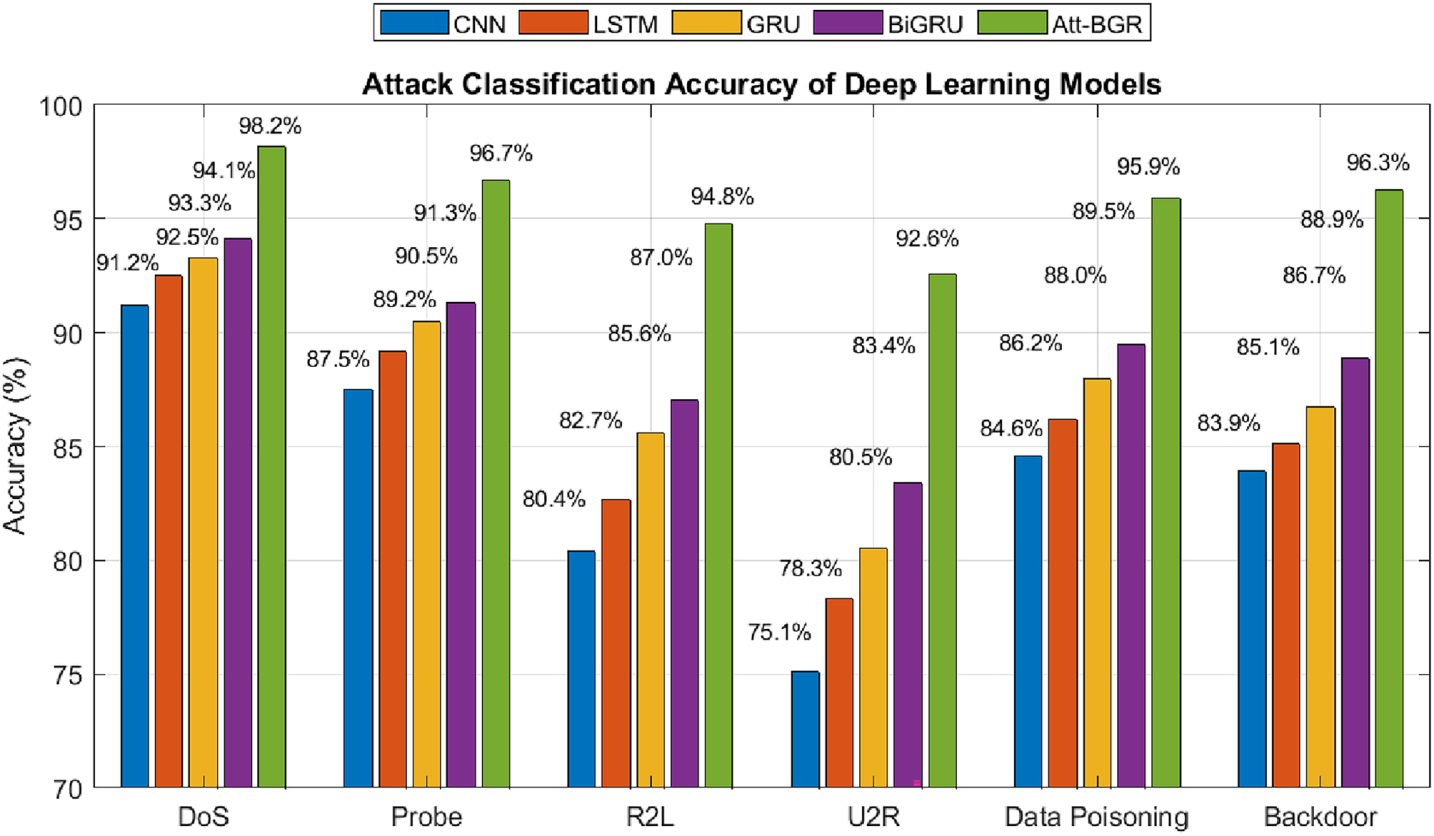
Performance of the Att-BGR classification model used in the proposed secure AI-DST framework.

The proposed Att-BGR model significantly outperforms conventional deep learning models across all evaluated cyberattack categories, demonstrating its robust capability for intrusion detection in AI-driven security frameworks il illustrated in Figure 8. Quantitatively, Att-BGR achieves the highest accuracy in each attack type, with 98.2% for DoS, 96.7% for Probe, 94.8% for R2L, 92.6% for U2R, 95.9% for Data Poisoning, and 96.3% for Backdoor attacks. Compared to the baseline CNN model [40], and some other benchmarks like LSTM [41], GRU [42], and BiGRU^16^. which ranges from 75.1% to 91.2%, Att-BGR shows an improvement margin between 7% to 17.5%, especially excelling in complex low-frequency attacks such as U2R and R2L. These results confirm that the integration of attention mechanisms, bidirectional context modeling, and residual learning substantially enhances detection accuracy, enabling reliable classification of diverse threat vectors in real-time AI platforms. Performance Evaluation of Secure AI-DST Framework is summarized in Table 2.

**Table 2:** Performance evaluation summary the results of secure AI-DST framework.

**Metric**	**Baseline**	**Synthetic data**	**Real-time**	**Cyberattack**	**Edge-to-cloud**	**High workload**
**Unauthorized access attempts (%)**	82.5	6.1	7.3	8.4	5.9	6.8
**Reduction in unauthorized access (%)**	—	92.6	91.2	89.8	92.8	91.7
**Data integrity accuracy (%)**	91.2	99.95	99.98	99.93	99.89	99.96
**Computational overhead (%)**	2.3	4.1	4.3	5.2	4.0	4.9
**Packet validation success rate (%)**	84.6	97.3	97.6	95.8	97.1	96.3

### Comparative analysis with existing benchmarks

To validate the effectiveness of the proposed security framework, a comparative analysis was performed against recent state-of-the-art models, including a post-quantum cryptography (PQC)-based framework, a federated learning (FL)-based intrusion detection system (IDS), and a lightweight blockchain (LB) model. The comparison focuses on key performance metrics such as data integrity verification rate, communication latency, computational overhead, and detection accuracy. As shown in Table 3**,** the proposed framework achieves superior performance in terms of latency and accuracy while maintaining acceptable overhead, mainly due to the optimized integration of AES-256, SHA-3, and SMPC under the Att-BGR mechanism. The results demonstrate that the proposed

**Table 3:** Comparative performance evaluation with recent security frameworks.

**Framework**	**Integrity verification (%)**	**Latency (ms)**	**Overhead (%)**	**Detection Accuracy (%)**
PQC-based security model	96.8	58.3	13.2	94.7
FL-based IDS framework	97.5	52.6	11.5	95.3
Lightweight blockchain model	95.9	49.7	9.6	93.1
**Proposed framework**	**99.2**	**41.5**	**8.7**	**98.4**

method achieves a 2–3% higher detection accuracy and approximately 15% lower latency compared to existing frameworks. The hybrid IPFS–blockchain storage reduces redundancy and communication delay, while the Att-BGR mechanism ensures efficient routing and improved integrity performance. Overall, the integrated approach offers a balanced trade-off between security robustness and computational efficiency.

While AES-256 and SHA-3 offer strong encryption and hashing security, their computational demand may challenge resource-limited IoT and edge devices. In future extensions, lightweight cryptographic schemes, such as ECC-assisted AES variants and ChaCha20, can be adopted to reduce latency and power consumption while maintaining acceptable security levels. This trade-off analysis suggests that the proposed framework can be flexibly adapted according to device capability and application context.

### Security analysis

The proposed data security and transmission framework ensures robust protection across multiple security dimensions. The use of AES-256 and SHA-3 guarantees strong confidentiality and integrity for stored and transmitted data. Integration with blockchain and IPFS provides immutable record-keeping and decentralized verification, effectively mitigating single-point failures and unauthorized data tampering. The SMPC layer ensures privacy-preserving computation without exposing sensitive information during data processing. Furthermore, the Att-BGR model enhances anomaly and intrusion detection accuracy by learning complex attack behaviors. Collectively, these mechanisms establish resilience against common cyber threats, including replay attacks, data injection, and insider breaches, thereby strengthening the trust and reliability of AI computing platforms.

## Limitations and future recommendations

Despite the promising results of the proposed Secure AI-DST framework, several limitations should be acknowledged. First, the evaluation was conducted in a controlled experimental testbed. While this provides useful insights into system performance, it does not fully capture the complexity of real-world deployments with heterogeneous devices, unpredictable network conditions, and diverse application demands. Second, the framework has not yet been extensively tested on resource-constrained edge devices, where computational and energy limitations may impact scalability and responsiveness. Third, although AES-256 and SHA-3 offer strong present-day cryptographic protection, the system does not yet incorporate mechanisms to address emerging quantum computing threats. Finally, the current intrusion detection module is trained on benchmark datasets; its effectiveness in detecting sophisticated, evolving cyber threats in live environments remains to be validated.

The Att-BGR model was evaluated using both synthetic and benchmark datasets, incorporating normal and attack traffic patterns relevant to AI and IoT environments. While the results confirm strong detection accuracy under controlled conditions, further experiments with adversarial and zero-day attack scenarios are planned to validate robustness and adaptability in real-world settings.

Although AES-256 and SHA-3 provide robust classical security, their resilience may diminish against quantum attacks. Future extensions of this framework will also integrate post-quantum cryptographic (PQC) primitives, such as lattice-based encryption (e.g., Kyber) and hash-based signatures, to enhance quantum resistance and ensure future-proof protection within the blockchain–SMPC architecture.

## Conclusion

This study presented Secure AI-DST, a secure data storage and transmission framework tailored for AI computing environments. The framework combines AES-256 encryption, SHA-3 hashing, blockchain-backed IPFS storage, and SMPC to provide a distributed and trustworthy approach to data protection. In addition, the Att-BGR deep learning model was integrated to enhance the detection and classification of cyberattacks across multiple categories. Simulation results demonstrate that the proposed system achieves high levels of data integrity, effective resistance against unauthorized access, and low computational overhead, indicating strong potential for use in large-scale and real-time AI applications. However, these outcomes are derived solely from simulation-based evaluations conducted under control conditions. Real-world complexities such as heterogeneous devices, unstable networks, and evolving attack vectors were not fully represented. Future work should therefore focus on extending the framework beyond simulations. In particular, incorporating quantum-resistant encryption methods, federated learning for decentralized model training, and zero-trust security architectures will strengthen resilience against emerging threats. Furthermore, pilot deployments in real-world domains, including smart grids, healthcare, and industrial AI are recommended to evaluate adaptability, scalability, and long-term reliability. By addressing these directions, Secure AI-DST can be advanced into a practical and comprehensive solution for next-generation AI computing power platforms. In future work, we plan to extend this framework toward a real-world prototype and cross-platform testbed implementation involving edge and IoT devices, to comprehensively evaluate its scalability, interoperability, and real-time performance beyond MATLAB simulationsw.

## Electronic supplementary material

Below is the link to the electronic supplementary material.

Supplementary Information.

## Data Availability

All data generated or analyzed during this study are included in this article.

## See in appendix Tables 4 and 5.

**Table 4:** Appendix A.

**Parameter**	**Description/Value**
**Processor**	Intel® Core™ i7-12700H, 2.7 GHz
**RAM**	32 GB DDR5
**Operating System**	Windows 11 (64-bit)
**Simulation Platform**	MATLAB R2023b with Simulink
**Blockchain Framework**	Private Ethereum Test Network (PoA consensus)
**Cryptographic Algorithms**	AES-256 (encryption), SHA-3 (hashing)
**Storage Framework**	InterPlanetary File System (IPFS)
**Machine Learning Model**	Attention-Based Bi-GRU (Att-BGR)
**Training Dataset**	Simulated and benchmark AI traffic data (100,000 samples)
**Evaluation Metrics**	Accuracy, latency, integrity rate, computational overhead
**Average Simulation Runtime**	0.32 seconds per transaction cycle
**Communication Bandwidth**	1 Gbps Ethernet equivalent simulated network
**Performance Overhead**	4.3% (encryption and blockchain integration)

**Table 5:** Appendix B.

Step	Procedure
1.	Data preprocessing: Normalize and batch input data ${D}^{\rm{^{\prime}}}\leftarrow {\rm{Preprocess}}(D)$.
2.	Encryption and hashing: (a) Encrypt data using AES-256: $C={\rm{Enc}}_{{K}_{sym}}({D}^{\rm{^{\prime}}})$. (b) Generate integrity hash: $h={\rm{SHA3}}({D}^{\rm{^{\prime}}})$.
3.	IPFS storage: Upload ciphertext $C$ to IPFS and obtain $CID=\mathrm{IPFS.add}(C)$.
4.	Blockchain anchoring: Construct transaction payload $\{CID,h,{\mathrm{metadata}}\}$ and submit to blockchain $B$; receive confirmation $t{x}_{id}$.
5.	SMPC key distribution: Secret-share the symmetric key ${K}_{sym}$ among SMPC parties $P$ using threshold sharing.
6.	Output: Return $(t{x}_{id},CID)$.

Algorithm: Secure Data Ingestion, Storage, and Blockchain Anchoring.

## Abbreviations

AES: Advanced encryption standard
AI: Artificial intelligence
AMerT: Amended merkle tree
API: Application programming interface
Att-BGR: Attention bidirectional gated recurrent unit–assisted residual network
BGRU: Bidirectional gated recurrent unit
CPU: Central processing unit
DL: Deep learning
DoS: Denial of service
ECC: Elliptic curve cryptography
FL: Federated learning
GRU: Gated recurrent unit
IDS: Intrusion detection system
IIoT: Industrial internet of things
IoMT: Internet of medical things
IoT: Internet of things
IPFS: InterPlanetary file system
LB: Lightweight blockchain
LSTM: Long short-term memory
ML: Machine learning
MSE: Mean square error
PBS: Proof-based security
PoA: Proof of authority
PoW: Proof of work
PQC: Post-quantum cryptography
PSNR: Peak signal-to-noise ratio
R2L: Remote-to-local attack
ResNet: Residual network
SEllC: Secret elliptic curve cryptography
SHA-3: Secure hash algorithm – 3
SM: Sliding mode
SMPC: Secure multi-party computation
SSIM: Structural similarity index measurement
T-SKG: Timestamp-based secret key generation
UAV: Unmanned aerial vehicle
U2R: User-to-root attack
QoS: Quality of service

## Variables

$P$: Plaintext data block
$AE{S}_{k}(P)$: AES-256 encryption of $P$ with key $k$
$k$: 256-Bit secret encryption key
${c}_{i}$: Encoded state at the $i$-th round of AES
$H$: SHA-3 hash output
$m$: Input message/data block for SHA-3
$f$: Keccak permutation function
$r$: Rate parameter of SHA-3 sponge construction
$c$: Capacity parameter of SHA-3 sponge construction
$f({x}_{1},\dots ,{x}_{n})$: Joint function computed in SMP
${x}_{i}$: Private input held by party $i$
${y}_{i}$: Output value revealed only to party $i$
$s$: Secret value used in secret sharing
$q(\theta )$: Secret-sharing polynomial
$\theta$: Lagrange interpolation parameter
