# Supplementary material for: Data security storage and transmission framework for AI computing power platforms
Source: Sci Rep. 2026 Jan 2;16:2230. doi: 10.1038/s41598-025-31786-5 (PMC12815940; doi:10.1038/s41598-025-31786-5)
Supplement: Supplementary file 1 — Supplementary Information. [file 41598_2025_31786_MOESM1_ESM.docx]

**Appendix B (Revised): Formal Theoretical Model, Algorithm, and Complexity Analysis**

**B.1 Mathematical Preliminaries**

Let $D=\{d_{1},d_{2},\ldots,d_{n}\}$denote the batched IoT data samples after preprocessing.
Let the symmetric encryption function be:

$$C=\text{Enc}_{K}(D)$$

where $K$is the session key.
The integrity hash is computed as:

$$h=\text{SHA3}(D)$$

Let the IPFS content identifier be:

$$CID=\text{IPFS.add}(C)$$

Let the blockchain anchoring function be:

$$\tau=\text{Anchor}(CID,h,\eta)$$

where $\eta$is metadata.

SMPC key distribution uses threshold sharing:

$$K\to\{K_{1},K_{2},\ldots,K_{m}\},\text{with threshold }t$$

**B.2 Lemmas, Theorems, and Proofs**

**Lemma 1: Correctness of Encryption–Hash Pipeline**

For a deterministic hash function $H$and a secure symmetric cipher $E$, the pair $(C,h)$uniquely represents the original data batch $D$.

**Proof:**
The encryption function $\text{Enc}_{K}(\cdot)$is injective under a fixed key $K$.
The hash $h=H(D)$uniquely maps $D$to a fixed digest due to collision resistance.
Thus, any change in $D$results in either a different ciphertext or a different hash value.
Hence, $(C,h)$uniquely identifies $D$.

**Theorem 1: Integrity Preservation Under IPFS–Blockchain Anchoring**

If the IPFS CID and hash $h$are both stored on-chain, any tampering of stored data $D$or its ciphertext $C$is detectable.

**Proof:**
Tampering with $C$changes its IPFS hash, generating a new CID'. Since $CID^{'}\neq CID$, verification fails.
Tampering with $D$changes $h=H(D)$to $h^{'}\neq h$.
Because both $(CID,h)$are recorded immutably on-chain, mismatched values reveal the tampering.

**Lemma 2: Confidentiality of SMPC-Based Key Sharing**

Given a threshold $t<m$, no subset of fewer than $t$parties can reconstruct the session key $K$.

**Proof:**
Based on Shamir’s Secret Sharing, each fragment $K_{i}$is an evaluation of a polynomial of degree $t-1$.
Fewer than $t$points cannot reconstruct the polynomial, hence $K$remains confidential.

**Theorem 2: Non-Repudiation and Immutability of Anchored Transactions**

Once $CID$and $h$are committed to a blockchain with PoA consensus, neither the sender nor the network operator can alter the record.

**Proof:**
In PoA networks, blocks are signed by authorized validators and stored immutably.
Changing any transaction would require re-signing the block or regenerating the chain, which is computationally infeasible without validator compromise.
Thus, anchored records provide non-repudiation and immutability.

**B.3 Updated Algorithm with Embedded Theorems**

**Algorithm 1: Secure Data Ingestion, Storage, and Blockchain Anchoring**

| **Step** | **Procedure** |
| --- | --- |
| **1. Data Preprocessing** | Normalize raw IoT data: $D^{'}=\text{Preprocess}(D)$. |
| **2. Encryption** | Compute ciphertext using AES-256: $C=\text{Enc}_{K}(D^{'})$. (Correctness ensured by Lemma 1). |
| **3. Integrity Hashing** | Compute hash: $h=\text{SHA3}(D^{'})$. |
| **4. IPFS Storage** | Upload $C$to IPFS: $CID=\text{IPFS.add}(C)$. Integrity guaranteed by Theorem 1. |
| **5. Blockchain Anchoring** | Submit transaction: $\tau=\text{Anchor}(CID,h,\eta)$. Immutability from Theorem 2. |
| **6. SMPC Key Distribution** | Distribute key using threshold sharing: $K\to\{K_{i}{\}}_{i=1}^{m}$. Confidentiality ensured by Lemma 2. |
| **7. Output** | Return verification tuple $(CID,h,\tau)$. |

**B.4 Complexity and Cost Analysis**

**1. Computational Complexity**

- **AES-256 encryption**: $O(n)$
- **SHA-3 hashing**: $O(n)$
- **IPFS hashing and chunking**: $O(n\log n)$
- **Blockchain transaction creation**: $O(1)$
- **SMPC key sharing**: $O(t)$

**Total runtime complexity:**

$$O(n\log n)$$

**2. Communication Complexity**

- IPFS upload: proportional to data size → $O(n)$
- Blockchain transaction: constant size → $O(1)$
- SMPC distribution: $O(m)$

**3. Performance & Cost Summary**

Measured using the system in Appendix A:

- **Transaction execution time:** 0.32 s
- **Overhead of encryption + blockchain anchoring:** 4.3%
- **Throughput:** 1 Gbps network link
- **IPFS retrieval latency:** sub-second for batches $<5$MB

These results validate the practicality of the proposed method under realistic system constraints.
